# Machine Learning-Based Analysis of Emotional Responses to Food Labels: A Case Study of Thai Young Adults

**DOI:** 10.3390/bs16050742

**Published:** 2026-05-10

**Authors:** Apsorn Sattayakhom, Waluka Amaek, Phanit Koomhin

**Affiliations:** 1 School of Allied Health Sciences, Walailak University, Nakhonsithammarat 80160, Thailand; apsorn.sa@wu.ac.th; 2 Center of Excellence in Innovation of Essential Oil and Bioactive Compounds, Walailak University, Nakhonsithammarat 80160, Thailand; 3 School of Information Studies, Walailak University, Nakhonsithammarat 80160, Thailand; waluka.am@wu.ac.th

**Keywords:** food label design, circumplex model of emotion, machine learning, sensory

## Abstract

Understanding the emotional drivers of consumer choice is critical for effective food packaging design. This study proposes a novel ‘Emotion–AI Framework’ to decode consumer responses to ten processed fish product labels using the circumplex model of emotion. Explicit emotional responses and purchase intentions were collected from 100 participants, and unsupervised machine learning (K-Means clustering) successfully classified consumers into three distinct segments (Enthusiasts, Passives, and Rejectors) strictly based on their multidimensional emotional profiles. Furthermore, a supervised Random Forest regression model, coupled with permutation feature importance, revealed that aggregated emotional states (specifically the low-arousal/pleasant and high-arousal/unpleasant quadrants) are the dominant drivers of purchase intention. Crucially, these emotional states significantly outperformed the direct impact of physical label attributes. The findings demonstrate that integrating theoretical emotional models with predictive machine learning provides robust, data-driven insights for the food industry, enabling the optimization of product labels to evoke targeted affective states and maximize consumer acceptance.

## 1. Introduction

The global food processing market is undergoing continuous growth, with projections indicating that demand will increase at a CAGR of approximately 11.82% by the year 2030 (Singh, 2025). This surge is primarily driven by global population growth and increasing urbanization, elevating the demand for processed food products. In this highly competitive market, effective packaging design is essential for attracting consumers. Consequently, the design of both front-of-package and back-of-package labels is crucial for conveying vital information about a product’s characteristics and key components to consumers (Hawley et al., 2013). For today’s increasingly health-conscious consumers, a significant relationship exists between food labels and consumption choices, highlighting the importance of reading product information, particularly nutritional facts (Gineikiene et al., 2017). Research confirms that specific packaging design features, such as layout and color schemes, significantly influence consumer visual processing and attention to nutritional information (Antunez et al., 2013).

In recent years, neuromarketing methods have advanced significantly, drawing insights from consumer behavior, product preference, and neuroscience to design labels that effectively capture initial consumer attention (Meyerding, 2019). However, after this initial attraction, the consumer transitions to a conscious cognitive process to evaluate the information presented on the product label. Beyond being a sensory purchaser, this conscious purchaser assesses the product’s perceived benefits, value, and likability (Booth & Freeman, 2014). Recent reviews emphasize that traditional hedonic acceptance testing rarely predicts food choice behavior accurately, as human decision-making is heavily driven by emotional processing (Low et al., 2022). Consequently, measuring emotional responses, specifically through explicit self-reports that have been shown to effectively discriminate between food stimuli, has become essential for understanding consumer behavior beyond merely liking a product (Jaeger et al., 2021; Low et al., 2022; Moranges et al., 2021). Measuring emotions is no longer merely a supplementary tool but has become central to comprehensively understanding the consumer food experience (Meiselman, 2021). The intricate relationship between sensory perception and food choice behavior serves as a critical mechanism driving the food industry (Kaneko et al., 2018). To analyze this conscious evaluation, explicit measurement techniques such as self-reported questionnaires have been utilized effectively within the food industry for decades. In parallel, implicit measurement techniques such as automated facial expression analysis, heart rate variability, and electroencephalography offer a complementary approach by capturing unconscious reactions. While implicit measures are valuable for contextual analysis, recent perspectives suggest that explicit self-reports are often more sensitive in discriminating specific product attributes (de Wijk & Noldus, 2021). Notably, a consumer’s response to sensory stimuli is subject to significant variability due to ethnic, cultural, and physiological differences (Shavitt & Barnes, 2020). Building upon this, recent evidence in gastronomy underscores the importance of sensory and emotional profiling in decoding the influence of emotional perception and consumer acceptance on specific attributes (J. Silva et al., 2025). While these differences are well-documented, a scholarly gap remains in determining how these diverse emotional perceptions can be systematically translated into data-driven design rules for specific food categories.

Advancements in machine learning technology enable more profound extraction of knowledge from complex consumer data (Fuentes et al., 2020; Shrirame et al., 2020), and current research is increasingly transitioning from traditional linear statistical methods toward machine learning (ML) to effectively decode emotional data characterized by inherent complexity and high variability (D. Lee et al., 2025). For instance, Random Forest models have been successfully utilized to predict packaging features that drive consumer emotional responses (Y. J. Lee et al., 2025). Despite these advancements, a significant gap remains in applying ML techniques to derive specific design rules tailored to diverse food product categories. Furthermore, recent studies have successfully employed ML to assess consumer buying and paying preferences for labeled products (Shen et al., 2021) and even utilized optical character recognition to technically decode food labels to gain better health insights (T. Silva et al., 2024). This evolution aligns with the emerging paradigm of “digital sensory science”, where integrating cutting-edge technologies is essential for decoding intricate consumer perceptions and guiding the future directions of food science (D. Lee et al., 2025). Central to this approach is feature engineering, the process of transforming qualitative food sensory attributes (e.g., visual cues and texture descriptors) into structured data representations. By systematically encoding these attributes, researchers can employ ML algorithms, such as Random Forest, to detect complex, non-linear patterns in consumer preferences while efficiently managing high-dimensional affective data using unsupervised clustering techniques. Both unsupervised and supervised learning have demonstrated remarkable potential to derive insights from explicit measures, which are often more cost-effective than integrated biometric instrumentation (de Wijk & Noldus, 2021; Low et al., 2022; Shen et al., 2021). This is particularly relevant because emotion itself is a complex process involving both conscious and unconscious systems that influence consumer behavior and cognitive appraisals (Achar et al., 2016). In food product evaluation, participants consistently exhibit a tendency to assign higher ratings to pleasant emotions than to unpleasant ones, a well-documented phenomenon widely recognized in food emotion research (Desmet & Schifferstein, 2008). Furthermore, understanding the structure of the affective lexicon is crucial for accurately distinguishing between psychological emotions (mental states) and physiological conditions (physical states) (Ortony et al., 1987). The circumplex model of emotion provides a robust framework for understanding these responses and organizing them into emotional quadrants along the valence and arousal dimensions (Russell, 1980). Despite the established utility of this model, there is a lack of structured frameworks linking specific label attributes to these emotional quadrants through predictive analytics. The current literature often presents a known understanding of general emotional influence, but there is a missing link regarding the precise drivers of purchase intention in specific, highly competitive food categories. Processed fish products were deliberately selected as the focal stimuli for this study. This category represents a highly saturated market where the core products are often physically similar, forcing consumers to rely heavily on diverse packaging cues—such as visual product forms, preservation media, and health claims—to make rapid, emotion-driven purchasing decisions. Consequently, it serves as an ideal and highly relevant model for isolating the specific impact of label attributes on consumer emotional states.

This study addresses this gap by proposing a novel, integrated methodological framework that bridges the divide between theoretical emotional modeling and practical industrial application. By utilizing a suite of ML techniques, we seek to move beyond incremental observations and provide actionable design insights. To clarify the scholarly contribution of this workflow, this research is guided by the following hypotheses and research questions:

**Hypotheses (H)**

- **H1**. *Emotional states characterized by positive valence (regardless of arousal intensity) act as the primary drivers of purchase intention, serving as the critical response mechanism through which physical label attributes exert their influence.*
- **H2**. *Distinct food label attributes (e.g., product imagery vs. nutritional claims) are associated with measurable and different effects on the dimensions of valence and arousal.*

**Research Questions (RQs)**

- **RQ1**. *Which specific emotional quadrants (e.g., excitement vs. contentment) most significantly drive purchase intention in the context of processed fish product labels?*
- **RQ2**. *How do specific visual and informational attributes of a label act as the primary drivers of valence and arousal?*
- **RQ3**. *To what extent can an integrated ML workflow provide more actionable design insights compared with traditional statistical methods?*

Accordingly, the objective of this study is to establish a clear, data-driven methodology that can guide the design of food labels to better meet the emotional and behavioral needs of consumers. We applied the circumplex model to measure responses to processed fish product labels, utilized unsupervised machine learning (K-Means) for consumer segmentation, and employed supervised models (decision trees and Random Forest regression) to identify the key drivers of purchase intention.

## 2. Materials and Methods

### 2.1. Participants

One hundred participants (50 male; 50 female) with an average age of 20.57 ± 1.13 years were recruited in this study. The protocol was approved by the Human Research Ethics Committee of Walailak University (approval number WUEC-20-035-01), and written informed consent was obtained from all subjects prior to their participation. The inclusion criteria required participants to be healthy university students with no history of visual impairment or food allergies.

### 2.2. Stimuli

Ten processed fish product labels were used as stimuli. These labels featured varying attributes across four dimensions: meat shape, media, health benefit, and shelf life. Ten cards were presented to the participants, with the details described in Table 1. Detailed visual designs and attribute descriptions for all ten food labels are available in the Supplementary Materials.

### 2.3. Procedure

**Figure 1:**
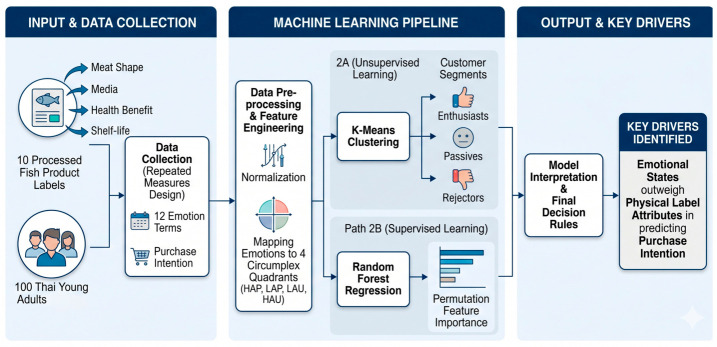
The proposed Emotion–AI research framework for decoding consumer responses to food labels.

The participants evaluated all 10 processed fish product labels and rated their emotional response using a 10 cm visual analog scale for 12 emotion terms (Figure 1). The 12 emotion terms utilized in this study were carefully selected and adapted from established affective lexicons fundamentally grounded in the Circumplex Model of Affect (Russell, 1980). This model proposes that affective experiences can be systematically organized along two orthogonal dimensions: valence (pleasant–unpleasant) and arousal (high–low). While certain negative terms in our list, such as ‘fatigue,’ ‘stress,’ and ‘lethargic,’ have occasionally been debated as reflecting physiological conditions rather than pure psychological emotions, their inclusion is highly relevant in the context of sensory science. In food evaluation, consumers often experience holistic affective states where mental feelings and physical sensations are deeply intertwined. Therefore, these terms were retained to capture the comprehensive affective responses evoked by the product labels. Furthermore, the questionnaire was administered in Thai. To ensure semantic equivalence and cultural validity, the emotion terms underwent a rigorous forward and backward translation process by bilingual experts, thereby ensuring that the intended affective meaning of each term was accurately conveyed to the local demographic. The presentation order of the 10 label cards was completely randomized for each participant to minimize carry-over and order effects, and these terms were subsequently mapped to the four quadrants of the circumplex model of emotion (Russell, 1980). Additionally, a 5-scale purchase intention score was collected for each evaluation (Teng et al., 2007).

### 2.4. Key Driver Analysis of Purchase Intention

To conduct the key driver analysis of purchase intention, a machine learning model was developed using the Random Forest regressor algorithm. The model’s target variable (Y) was the purchase intention score, and the predictor variables (feature matrix, X) were constructed from three primary data sources: (1) the four aggregated emotional quadrants (high arousal–pleasant (HAP), low arousal–pleasant (LAP), low arousal–unpleasant (LAU), and high arousal–unpleasant (HAU)); (2) the four product attributes (meat shape, media, health benefit, and shelf life); and (3) demographic information (gender). Categorical features were converted into a numerical format using one-hot encoding. To ensure reproducibility, the Random Forest regressor was configured with 100 estimators (n_estimators = 100) and a fixed random state (random_state = 42). To prevent data leakage from repeated measures, we employed a 5-fold subject-grouped cross-validation strategy, and the model’s predictive performance was evaluated using root mean squared error (RMSE) and mean absolute error (MAE). Finally, Permutation Feature Importance (10 repetitions) was computed to robustly identify the key drivers, thereby addressing any potential multicollinearity among emotional variables.

### 2.5. Behavioral Consumer Segments

To identify behavioral consumer segments, an unsupervised machine learning approach was employed using the K-Means clustering algorithm. *Data preparation and feature engineering:* A consumer profile was engineered for each participant. This profile consisted of a 4-dimensional feature vector derived exclusively from the emotional quadrants: (1) HAP, (2) LAP, (3) LAU, and (4) HAU. “Purchase intention” was excluded from the clustering input to avoid circularity and instead used as an external validation metric. To ensure reproducibility and prevent data leakage, we implemented a strict machine learning pipeline using the scikit-learn library. Data preprocessing, including standard scaling, was performed within a pipeline to ensure that these transformations were fitted correctly. *Determination of optimal cluster number:* The elbow method was utilized to determine the optimal number of clusters (k) by running the K-Means algorithm for k values ranging from 1 to 10 and plotting the inertia. *Clustering and profile analysis:* Participants were segmented using the K-Means algorithm with the determined optimal k. The resulting clusters were then characterized by calculating the mean profile for each segment and examining the demographic (gender) distribution within each cluster. *Visualization:* Principal component analysis was used to reduce the 4-dimensional profile data into two principal components for visualization, thus allowing the distinct consumer segments to be plotted and inspected in a two-dimensional space.

### 2.6. Decision Tree for New Customer Segment

To generate profiles and classification rules for the identified consumer segments, a combination of unsupervised and supervised machine learning techniques was applied. *Consumer segmentation:* Participants were segmented using the K-Means clustering algorithm as described above. *Segment profiling and rule generation:* Following the segmentation step, a decision tree classifier was trained to generate a simple and interpretable set of classification rules for the discovered clusters. The 4-dimensional consumer profiles were used as the independent variables, and the resulting Cluster ID was used as the dependent variable. Pre-pruning was applied to the decision tree (max_depth = 3) to prevent overfitting and enhance interpretability. The final output was visualized as a decision tree diagram to illustrate the classification logic. The decision tree was used for descriptive purposes to visualize cluster boundaries. The reported accuracy indicates the model’s ability to represent the K-Means logic rather than its predictive performance on new subjects.

### 2.7. Statistical Analysis

Descriptive statistics were used to describe the shape of the data, and general data were reported as means ± SDs. Data analysis and machine learning modeling were performed using the Python programming language (version 3.12.12) on the Google Collaboratory platform, and the analysis utilized the *pandas* (version 2.2.2) and *numpy* (version 2.0.2) libraries for data manipulation, *scikit-learn* (version 1.6.1) for machine learning algorithms (clustering, regression, decision trees, and validation metrics), and *matplotlib* (version 3.10.0) and *seaborn* (version 0.13.2) for data visualization. All figures were generated directly from the Python script.

## 3. Results

### 3.1. Overall Consumer Emotional Responses

**Figure 2:**
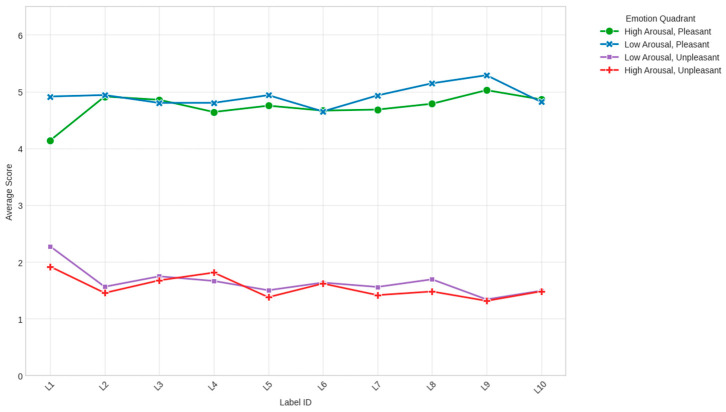
The overall emotional response of consumers to ten different processed fish product labels, which varied in product characteristics, health benefits, and shelf life. The analysis utilized twelve emotion terms derived from Russell’s circumplex model, which were aggregated into four distinct emotional quadrants.

Based on the responses from 100 participants, the quadrant with the highest average score was low arousal and pleasant (mean = 4.92), followed by high arousal and pleasant (mean = 4.73). The negative emotion quadrants scored significantly lower, with low arousal and unpleasant at a mean of 1.65 and high arousal and unpleasant at a mean of 1.56. Internal consistency analysis confirmed the reliability of these quadrants, with Cronbach’s alpha coefficients ranging from 0.84 (LAP/LAU) to 0.91 (HAU) (Figure 2).

### 3.2. Key Drivers of Purchase Intention: The Role of Emotion vs. Product Attributes

**Figure 3:**
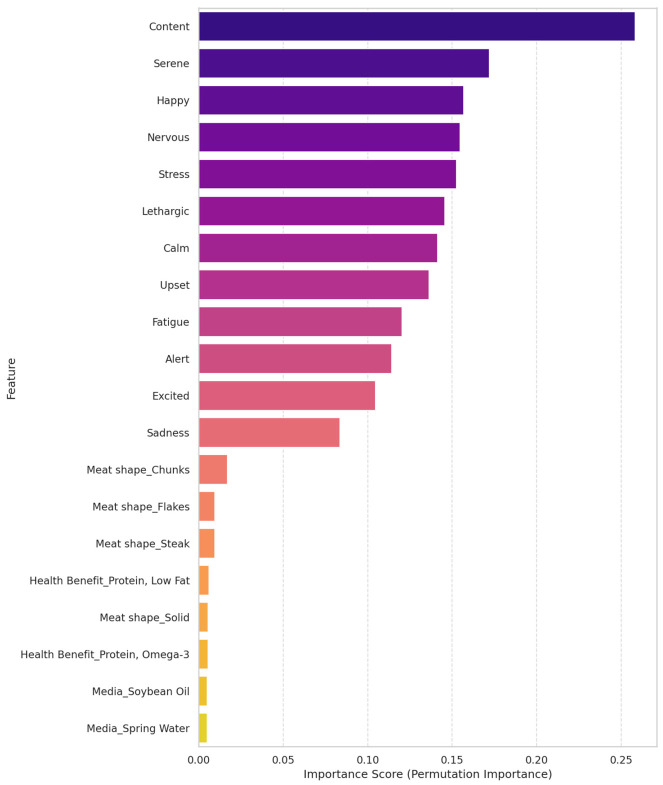
Key driver analysis of purchase intention derived from a Random Forest regressor model. The chart illustrates the relative importance of the 12 individual emotion terms and product attributes, highlighting the granular impact of specific emotional states on consumer decision-making.

**Figure 4:**
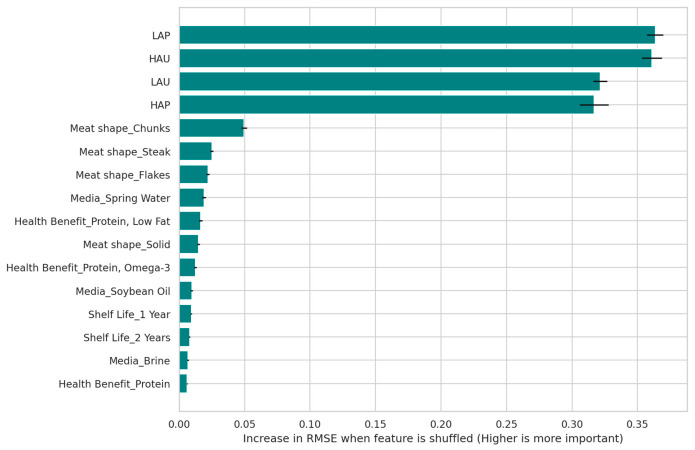
Key drivers of purchase intention identified via Permutation Feature Importance using the four aggregated emotional quadrants. The chart highlights the dominant predictive power of the LAP and HAU quadrants compared with physical product attributes. The horizontal black lines on the bars represent error bars, indicating the standard deviation of the importance scores across permutations.

The analysis using Permutation Feature Importance reveals that aggregated emotional quadrants exhibited the highest relative importance in predicting purchase intention within the model (Figure 3 and Figure 4). The Random Forest model achieved an RMSE of 0.83 ± 0.03 and an MAE of 0.67 ± 0.04, indicating robust predictive accuracy. Specifically, the LAP and HAU quadrants emerged as the top drivers, showing the highest importance scores of 0.363 and 0.361, respectively. In contrast, physical product attributes, such as meat shape and media, ranked significantly lower, while the most influential physical attribute, “Meat shape_Chunks”, achieved an importance score of only 0.050.

To identify the determinants of purchase intention, we first analyzed the impact of the 12 individual emotion terms along with product attributes. As shown in Figure 3, positive low-arousal emotions such as “Content”, “Serene”, and “Happy” appeared as the top drivers. However, the importance scores for these individual terms were relatively low (max < 0.12), suggesting that the predictive signal was diluted across semantically similar terms (multicollinearity).

To address this and enhance model robustness, we aggregated these terms into the four theoretical circumplex quadrants (HAP, LAP, LAU, and HAU). The results from this aggregated model (Figure 4) revealed a substantial increase in predictive power. The LAP quadrant emerged as the dominant driver, with an importance score of 0.363, approximately three times stronger than any single raw emotion. This confirms that the aggregated emotional state is the primary predictor of purchase intention, far outweighing the impact of physical product attributes like media or meat shape.

The comparison reveals that using the four emotional quadrants provides a more strategic and interpretable overview of the key drivers. As shown in Figure 4, the permutation importance analysis utilizing aggregated quadrants revealed a distinct hierarchy of drivers. The emotional quadrants emerged as the dominant predictors, with LAP and “high arousal, unpleasant” (HAU) showing the highest impact on model error (importance approximately 0.36). In contrast, physical attributes such as “meat shape” and “media” showed significantly lower importance scores (<0.05), indicating that while design attributes trigger emotions, it is the emotional state itself that directly drives purchase intention. This finding is further illuminated when compared with Figure 3, where the predictive power was distributed across constituent raw emotions (e.g., content, serene, and calm) with much lower individual importance scores. This confirms that aggregating raw emotions into theoretically grounded quadrants effectively reduces noise and highlights the overarching emotional states, defined by the dimensions of valence and arousal, that are most critical to driving purchase intention.

### 3.3. Consumer Segmentation and Rule Extraction

**Figure 5:**
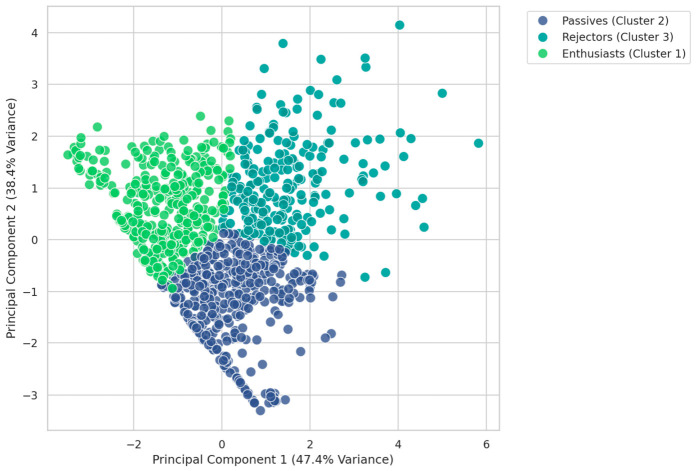
Customer segmentation based on emotional profiles visualized using principal component analysis (PCA). The scatter plot illustrates three distinct consumer clusters identified by K-Means clustering: “Enthusiasts” (Cluster 1, green), “Passives” (Cluster 2, purple), and “Rejectors” (Cluster 3, teal). The first two principal components together explain 85.8% of the total variance (PC1 = 47.4%; PC2 = 38.4%), demonstrating robust separation of consumer groups based on their aggregated emotional responses (HAP, LAP, LAU, and HAU).

The analysis identified three distinct consumer segments crucial for product label development: (1) Enthusiasts (Cluster 1), who exhibit a high probability of purchasing; (2) Passives (Cluster 2), who represent a potential conversion opportunity; and (3) Rejectors (Cluster 3), who demonstrate a clear refusal of the product (Figure 5). The identification of these behavior-based segments highlights the distinct consumer profiles present in the market.

To understand the specific impact of product attributes on these emotional states, descriptive profiling of the average emotion scores across different label designs was conducted. For the key pleasant quadrants, the “steak” and “flake” meat shapes consistently elicited the highest positive emotional responses. Conversely, the “chunks” meat shape was most frequently associated with negative emotional states, provoking the highest levels of unpleasant arousal. Other attributes, such as media type (soybean oil or brine), showed relatively minor variations compared with the dominant influence of meat shape. This descriptive analysis provides a clear blueprint of the attributes that enhance or detract from a positive emotional response, further confirming that evoking a calm and pleasant emotional state (LAP) is essential, while strongly avoiding product forms (like chunks) that trigger negative arousal acts as a major barrier to purchase intention.

**Figure 6:**
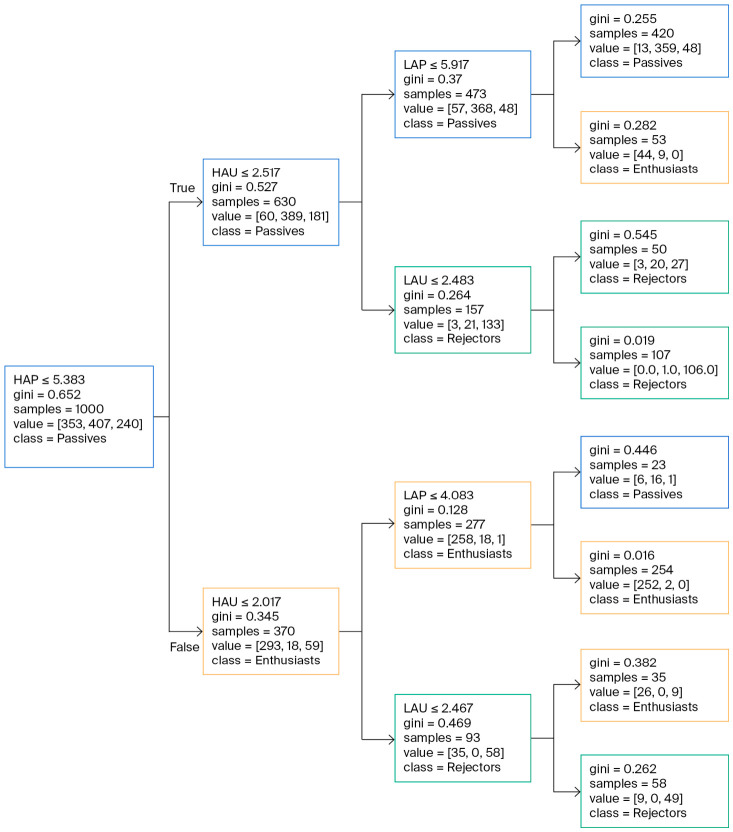
Decision tree classification rules defining consumer segments based on emotional profiles. The model utilizes hierarchical splitting of emotion scores to describe segment characteristics with 87.9% fit accuracy. The tree structure reveals distinct pathways: “Passives” are characterized by low arousal. “Enthusiasts” are characterized by high pleasantness, and “Rejectors” are characterized by high unpleasantness.

The classification rules derived from the decision tree are shown in Figure 6. As purchase intention was excluded from the clustering inputs to ensure methodological rigor, the model relies exclusively on emotional profiles to define the segments, achieving a classification accuracy of 87.90%. The decision tree identifies HAP as the most significant primary splitting variable. Participants with a high HAP score (>5.38) are predominantly classified as Enthusiasts, particularly when accompanied by low levels of negative arousal (HAU ≤ 2.02). Conversely, for participants with lower HAP scores (≤5.38), the model utilizes HAU as the critical secondary criterion. Those with higher negative arousal (HAU > 2.52) are classified as Rejectors, reflecting a clear emotional aversion. Meanwhile, participants characterized by both low HAP and low HAU (≤2.52) typically fall into the Passives segment, indicating a lack of strong emotional engagement, whether positive or negative.

## 4. Discussion

In addition to the tangible attributes of a product, the emotional response to a stimulus is a critical factor driving purchasing behavior. Importantly, while our study utilized explicit measures—a traditional technique capturing holistic affective states that encompass both psychological emotions and physiological conditions—we addressed its inherent analytical limitations by transitioning towards a machine learning (ML) paradigm. Recent advancements in sensory science highlight this shift towards artificial intelligence (AI) to handle the multidimensional complexity of consumer data (D. Lee et al., 2025).

Beyond the descriptive measurement of affective responses, the primary novelty and contribution of this research lie in the establishment of a structured ‘Emotion–AI Framework.’ While the influence of emotions on food choice is well-documented, this study transcends traditional approaches by integrating ML algorithms to systematically decode these complex human perceptions. Specifically, the application of K-Means clustering allowed for high-precision consumer segmentation based on multidimensional emotional profiles. Furthermore, consistent with recent reviews indicating that Random Forest (RF) models effectively prevent overfitting and offer high predictive accuracy in food quality studies (Y. J. Lee et al., 2025), our regression model identified key drivers of purchase intention with superior accuracy, achieving a robust RMSE of 0.83. To ensure statistical rigor, our study utilized Permutation Feature Importance as a key driver analysis tool. By decoupling the correlated effects of design attributes, this AI-driven framework revealed that emotional states (specifically valence and arousal) serve as the primary proximal drivers of purchase intention, effectively channeling the initial impact of physical product attributes into final consumer decisions. This integrated computational approach offers a significant advancement over traditional univariate statistical methods. While K-Means clustering efficiently partitions the high-dimensional affective data based on spatial proximity, the Random Forest regression model successfully captures the non-linear and intricate interactions between product attributes and emotional responses. Together, they provide a data-driven pathway for translating subjective emotional experiences into actionable design rules for the food industry.

Our findings strongly support the utility of the circumplex model as a feature engineering framework for consumer research. The analysis showed that using the 12 raw emotion terms resulted in a “masking effect”, where the importance of the emotional signal was distributed among semantically similar terms (e.g., calm, serene, and content), thereby lowering the apparent impact of any single variable. This aligns with the challenge of “noise” and “data leakage” in high-dimensional datasets often cited in ML applications. By aggregating these terms into the four theoretical quadrants (HAP, LAP, LAU, and HAU), we were able to reduce noise and multicollinearity, revealing that the emotional state itself (specifically LAP and HAU) is a dominant driver of purchase intention (importance > 0.30), far outweighing physical product attributes. This suggests that for machine learning applications in sensory science, theoretical aggregation yields more actionable and robust models compared with using high-dimensional raw data.

The results from this experiment reveal that the emotions elicited from viewing the label act as a vital bridge, translating the physical design attributes into actual purchase intention. Our findings align with the systematic review by Low et al. in 2022 (Low et al., 2022), who stated that visual cues (such as packaging) are potent drivers of the evoked emotions that directly influence expectations and purchase intention. Furthermore, while implicit measures exist, our study demonstrates the robustness of explicit emotional profiling when combined with advanced data science techniques to manage data heterogeneity (Moranges et al., 2021). Therefore, effective label design is not simply about including the best features but about how well those features can generate a positive emotional response in the consumer (Mora et al., 2018). Based on the attributes found to affect emotion in this study, it is recommended that marketing designs consider visual cues that promote “low arousal–pleasant” states such as steak or flakes meat shapes while noting that attributes like “chunks” tended to be associated with higher unpleasant arousal in this specific demographic (Bagozzi et al., 2016). Crucially, the utility of this “Emotion–AI” framework extends beyond the processed seafood category. The methodological pipeline established here, which links visual feature engineering with the circumplex model and predictive ML, serves as a scalable methodology for the broader food and beverage industry (Pearson, 2013). Manufacturers in other sectors, such as plant-based alternatives, functional beverages, and snack foods, can replicate this workflow to identify their own category-specific emotional drivers, thereby enhancing intuition-based design with data-driven precision.

The unsupervised learning analysis revealed a critical insight: the consumer market is not monolithic. While traditional clustering algorithms analyze customer segmentation (Kansal et al., 2018), our study leveraged K-Means clustering followed by a decision tree classifier to establish interpretable rules. The consumer base can be clearly divided into three distinct segments, each with a unique behavioral and emotional profile. This finding confirms that a one-size-fits-all strategy is unsuitable for this product, necessitating tailored approaches for each consumer segment. Critically, as purchase intention was excluded to ensure methodological rigor, we found that the HAP and HAU scores are the two most powerful discriminators for differentiating between these consumer groups. This implies that consumers categorize themselves primarily based on their distinct levels of excitement and displeasure.

This study was based on traditional explicit data. The strong discrimination between label designs observed in our study supports the view by de Wijk and Noldus (2021), who stated that explicit measures are highly effective for evaluating product-intrinsic sensory cues. However, as noted by D. Lee et al. (2025), explicit measures rely on subjective perception and may be influenced by cognitive biases or interindividual differences (de Wijk & Noldus, 2021; D. Lee et al., 2025). To translate these findings into actionable recommendations with the highest accuracy, a more comprehensive set of attributes should be analyzed. Future research should integrate “implicit” measurement techniques to complement these self-reports. For example, biometric technologies such as facial expression analysis (FEA) and electroencephalography (EEG) can quantify unconscious emotional responses, thereby providing a more objective validation of the reported “pleasant” or “unpleasant” states. Furthermore, purchasing behavior is influenced by a range of geographical and cultural factors (Torrico et al., 2019), and integrating these multimodal data sources, explicit surveys, digital label attributes, and implicit biometric signals into a unified AI framework would enable the development of “intelligent adaptive systems” capable of personalized product optimization, as envisioned in the latest sensory science trends. However, it is important to acknowledge that the emotional responses and preferences identified in this study (e.g., the preference for “steak” over “chunk” shapes) are observed within a specific demographic of young Thai adult consumers. Given that emotional associations with food packaging are deeply rooted in cultural and regional contexts, these findings should be interpreted as a preliminary case study, and future research across diverse cultural backgrounds and age groups is required to determine whether these design associations are consistent on a global scale.

This study acknowledges certain limitations regarding its external validity. First, the participants were primarily university students (mean age = 20.6 years), representing the young adult demographic. While this group is a key target market for ready-to-eat products, their preferences may not be fully generalizable to older consumers or other socioeconomic groups. Second, the use of simplified mock-up labels, while necessary to control for brand and price effects, may not fully capture the complexity of real-world purchasing decisions where brand loyalty plays a role. Future research should aim to replicate this framework with a broader demographic sample and incorporate commercial-grade packaging to validate these findings across diverse market segments. Furthermore, a methodological limitation of this study is the absence of pre-stimulus baseline emotion measurements. Since emotional reactivity varies individually, the lack of covariate adjustment for prior mood states limits the isolation of stimulus-induced affective shifts. Additionally, future frameworks should incorporate covariates such as participants’ specific dietary habits and prior brand familiarity, which may also significantly influence emotional responses. Finally, regarding the consumer segmentation methodology, the PCA diagram (Figure 5) indicated some degree of overlap between the identified clusters. To address this, future studies should explore alternative dimensionality reduction techniques, such as t-Distributed Stochastic Neighbor Embedding, along with advanced probabilistic clustering algorithms like Gaussian Mixture Models, to better evaluate cluster separation and uncover more complex underlying data structures.

## 5. Conclusions

This research demonstrates an integrated methodology combining the circumplex model of emotion with a suite of machine learning techniques to deconstruct consumer responses to processed fish product labels. By aggregating raw emotion terms into theoretical quadrants, we established a robust feature engineering framework that significantly enhances predictive power compared with traditional granular analysis.

We identified three distinct, behavior-based consumer segments from the key findings of the unsupervised learning analysis, namely Enthusiasts, Passives, and Rejectors, and generated interpretable decision rules for their classification. Furthermore, the supervised modeling (Random Forest regression) revealed a critical hierarchy in purchase drivers: the emotional state itself (specifically the “low arousal, pleasant” quadrant) acts as the primary determinant of purchase intention, functioning as the essential mechanism that links physical product attributes to consumer behavioral outcomes. Translating this into a design strategy, the analysis indicated that specific attributes, notably “steak” and “flake” meat shapes, are effective at eliciting these favorable calm and pleasant states, whereas the “chunks” shape acts as a significant barrier by triggering unpleasant arousal.

In conclusion, while the specific design preferences observed are contextual to the studied Thai demographic, this research demonstrates the efficacy of the Emotion–AI framework in decoding consumer sentiment. The primary contribution of this work lies in the methodological integration of the circumplex model of emotion with machine learning, thereby providing a blueprint for food designers to systematically evaluate emotional triggers in various markets.

## Figures and Tables

**Table 1:** Attributes of each label.

Label ID	Meat Shape	Media	Health Benefit	Shelf Life
L1	Chunks	Brine	Protein	1 Year
L2	Steak	Brine	Protein	1 Year
L3	Shredded	Brine	Protein	1 Year
L4	Chunks	Soybean Oil	Protein, Omega-3	2 Years
L5	Steak	Soybean Oil	Protein, Omega-3	2 Years
L6	Shredded	Soybean Oil	Protein, Omega-3	2 Years
L7	Chunks	Spring Water	Protein, Low Fat	2 Years
L8	Steak	Spring Water	Protein, Low Fat	2 Years
L9	Shredded	Spring Water	Protein, Low Fat	2 Years
L10	Solid	Soybean Oil	Protein, Omega-3	2 Years

Note: The presented labels are mock-up visual designs developed specifically for this study.

## Data Availability

The dataset generated and analyzed during the current study is publicly available in the Google Drive repository at [https://docs.google.com/spreadsheets/d/1xv099p3rzy6DtqomCRSI-3ROfy7ZyYjy/edit?usp=sharing] (accessed on 5 May 2026).

## Supplementary Materials

The following supporting information can be downloaded at https://www.mdpi.com/article/10.3390/bs16050742/s1; Figure S1: Optimal k determination (Elbow Method & Silhouette Score); Figure S2: Distribution of Purchase intention scores; File S2: Python analysis scripts for the machine learning pipeline.

## Abbreviations

The following abbreviations are used in this manuscript:

AI	Artificial Intelligence
CAGR	Compound Annual Growth Rate
EEG	Electroencephalography
HAP	High Arousal, Pleasant
HAU	High Arousal, Unpleasant
LAP	Low Arousal, Pleasant
LAU	Low Arousal, Unpleasant
MAE	Mean Absolute Error
ML	Machine Learning
PCA	Principal Component Analysis
RF	Random Forest
RMSE	Root Mean Squared Error
VAS	Visual Analog Scale
