# Supplementary material for: Machine Learning-Based Analysis of Emotional Responses to Food Labels: A Case Study of Thai Young Adults
Source: Behav Sci (Basel). 2026 May 10;16(5):742. doi: 10.3390/bs16050742 (PMC13203386; doi:10.3390/bs16050742)
Supplement: Supplementary file 1 [file behavsci-16-00742-s001.zip › Supplementary Materials_S1.pdf]

Supplementary Materials

Manuscript Title: Machine Learning-Based Analysis of Emotional Responses to Food Labels: A Case Study of Thai Young Adults

Authors: Apsorn Sattayakhom , Waluka Amaek and Phanit Koomhin

---

S1. Determination of Optimal Clusters for Consumer Segmentation

To determine the optimal number of consumer segments derived from emotional profiles (HAP, LAP, LAU, HAU), we employed K-Means clustering and validated the results using two internal validity indices: the Elbow Method (Inertia) and the Silhouette Coefficient.

Figure S1 illustrates the evaluation metrics for cluster counts (k) ranging from 2 to 10.

- **Elbow Method (Left Panel):** The inertia (within-cluster sum of squares) shows a distinct inflection point at k=3, suggesting that adding more clusters beyond this point yields diminishing returns in compactness.
- **Silhouette Analysis (Right Panel):** The average Silhouette Score peaks at k=3, indicating that the separation distance between resulting clusters is maximized and that the clusters are clearly defined.

Based on these converging lines of evidence, k=3 was selected as the optimal number of segments, which were subsequently labeled as Enthusiasts, Passives, and Rejectors.

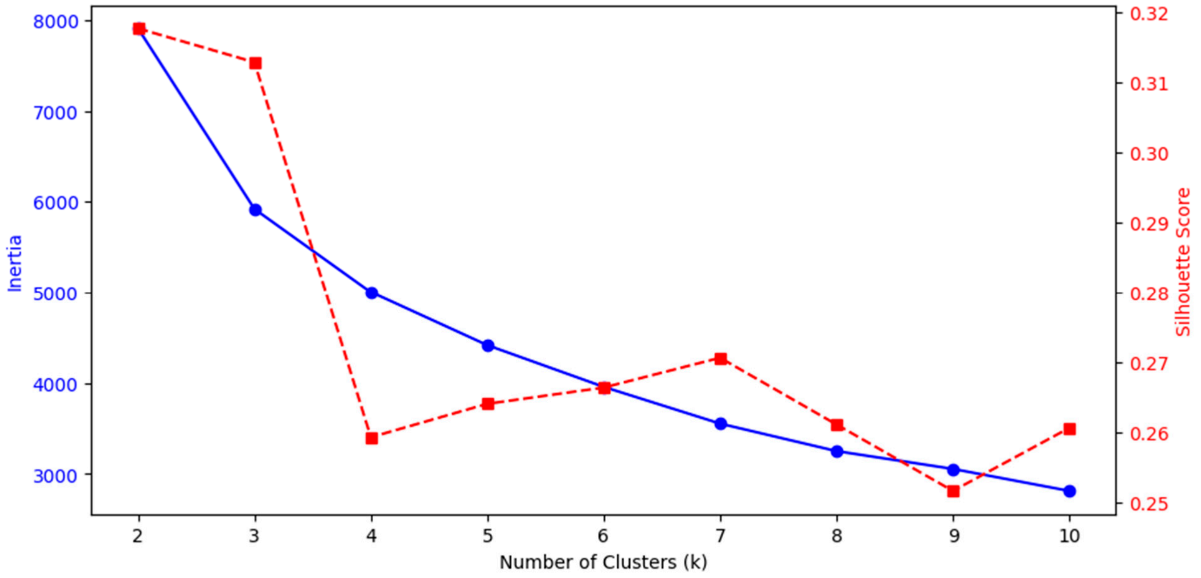

Figure S1. Validation of the optimal number of clusters. The Elbow Method (left) and Silhouette Scores (right) both support the selection of k=3 as the optimal solution for segmenting consumers based on emotional quadrants.

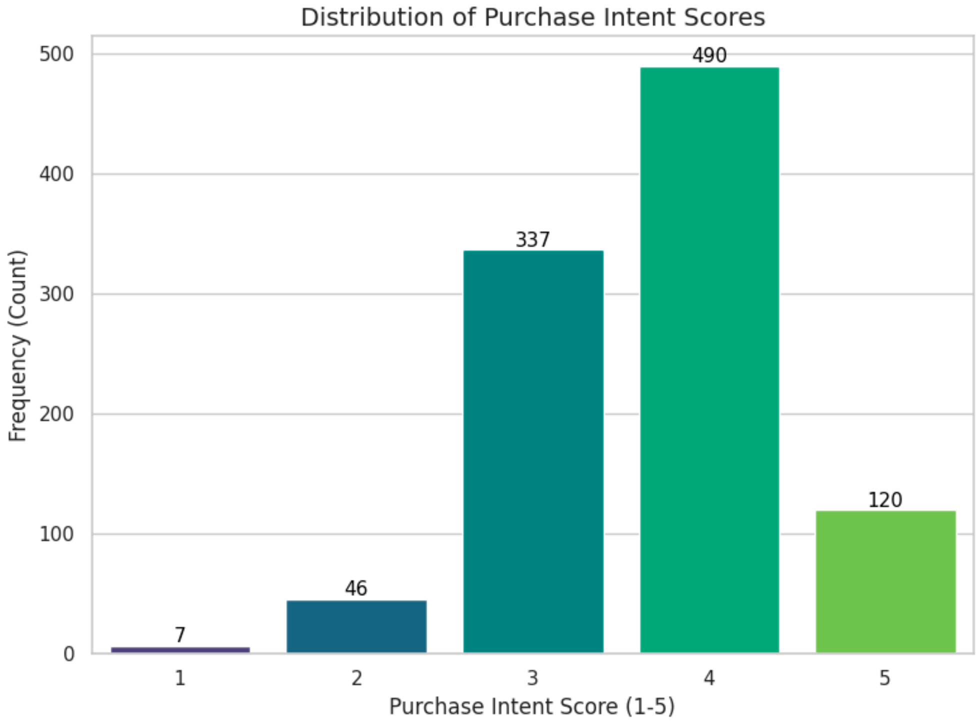

Figure S2. Distribution of Purchase Intent scores across all participants and stimuli (N=1,000 observations). The data shows a spread of responses across the 5-point scale, supporting the suitability of the dataset for regression analysis.
